# Collisions of deformable cells lead to collective migration

**DOI:** 10.1038/srep09172

**Published:** 2015-03-17

**Authors:** Jakob Löber, Falko Ziebert, Igor S. Aranson

**Affiliations:** 1 Institut für Theoretische Physik, Hardenbergstrasse 36, EW 7-1, Technische Universität Berlin, 10623 Berlin, Germany; 2 Physikalisches Institut, Albert-Ludwigs-Universität Freiburg, Hermann-Herder-Strasse 3, 79104 Freiburg, Germany; 3 Institut Charles Sadron, CNRS-UPR22, 23 rue du Loess, 67034 Strasbourg Cedex 2, France; 4 Materials Science Division, Argonne National Laboratory, 9700 S. Cass Avenue, Argonne, IL 60439, USA; 5 Engineering Sciences and Applied Mathematics, Northwestern University, 2145 Sheridan Road, Evanston, IL 60202, USA

## Abstract

Collective migration of eukaryotic cells plays a fundamental role in tissue growth, wound healing and immune response. The motion, arising spontaneously or in response to chemical and mechanical stimuli, is also important for understanding life-threatening pathologies, such as cancer and metastasis formation. We present a phase-field model to describe the movement of many self-organized, interacting cells. The model takes into account the main mechanisms of cell motility – acto-myosin dynamics, as well as substrate-mediated and cell-cell adhesion. It predicts that collective cell migration emerges spontaneously as a result of inelastic collisions between neighboring cells: collisions lead to a mutual alignment of the cell velocities and to the formation of coherently-moving multi-cellular clusters. Small cell-to-cell adhesion, in turn, reduces the propensity for large-scale collective migration, while higher adhesion leads to the formation of moving bands. Our study provides valuable insight into biological processes associated with collective cell motility.

## Introduction

While a significant effort was focused on understanding the mechanics, dynamics and motility of individual cells, the processes determining *collective* cell migration remain elusive to a large extent. There has been a body of experimental work on the motility of cells in monolayers, typically in the context of wound healing^1^,^2^. Collective motion of a few individual cells in a small adhesive spot, i.e., not in the context of tissue, was initiated in Ref. 3. Stimulated by the progress in designing patterned surfaces with controlled adhesive properties, it attracted considerable interest and was followed by detailed studies of collective cell motion in confined adhesive domains^4^,^5^,^6^. Studies on unbound substrates, as well as on domains with geometrical constraints, have been undertaken using various cell types like keratocytes and canine kidney cells^7^,^8^,^9^,^10^.

The key processes for single cell motility include acto-myosin dynamics^11^,^12^,^13^, and substrate-related adhesion dynamics^14^,^15^. A plethora of interactions emerge for collective cell motion, including the cells' deformability and polarization in response to the other cells, cell-cell adhesion, and signaling^16^,^17^,^18^,^19^. For example, comparisons of cancerous cells, exhibiting less inter-cellular adhesion, to healthy cells revealed that cell-cell adhesion critically affects collective cell behavior^5^,^20^. To characterize the propensity of cells to move collectively within a cell sheet, the notion of *plithotaxis*, the tendency of individual cells to migrate along the local orientation of the maximal principal stress, was introduced^21^,^22^. The migration velocity fields^2^ and the physical forces during collective cell migration^23^ have been experimentally characterized. However, predictive models relating these observations to the underlying physical and biochemical processes are not available to date.

There is a variety of modeling approaches to collective cell migration, including Vicsek-type models of self-propelled particles without^24^ or with inter-particle adhesion^25^,^26^,^27^,^28^, particle-based approaches^29^, lattice models^5^,^30^ and elastic spring models^31^ of epithelial spreading/wound healing, as well as phenomenological continuum theories focusing on various aspects^32^,^33^,^34^,^35^,^36^,^37^,^38^,^39^. All these approaches neglect the crucial dynamics associated with individual cell deformations (Refs. 40, 41 consider deformable elliptical particles, however allow only for the simplest shape deformations). The phase-field approach has been successfully applied to model the motility of individual cells^42^,^43^,^44^,^45^,^46^,^47^,^48^ and “active droplets”^49^,^50^. Recent work^48^ focused on the effects of cell nuclei and contact inhibition for the rotation of two cells on a micro-patterned spot, similar to the situation studied in Ref. 3.

Here we present a phase-field description of collective cell migration. The model captures the motility of up to a hundred cells (with present computer power). Our study reproduces salient features of cell-cell interactions and makes testable predictions on the role of cell density, cell-cell adhesion, and confinement on collective migration. It revealed that cell-cell adhesion has non-trivial implications: only a strong adhesion yields formation of large cell aggregates. Intermediate cell adhesion leads to transient multi-cell clusters and to an overall suppression of collective migration.

### Phase-field model

Our computational model is an extension of the phase-field model for an individual cell developed in Refs. 43, 45, 47 to multiple cells. Details are presented in Methods.

The model incorporates basic processes involved in substrate-based cell motility, such as protrusion via actin filament polymerization at the cell's leading edge, intermittent formation of adhesion sites to transfer momentum to the substrate, and detachment of adhesion complexes and myosin motor-driven contraction at the cell's rear. These mechanisms are cast into four continuous two-dimensional (2D) fields: the deformable and moving interface (the cell's membrane) is described by an auxiliary phase field *ρ*(*x*, *y*; *t*) governed by an overdamped diffusive motion, in a double-well model free energy that has minima for the two “phases” [inside the cell (*ρ* = 1) and outside the cell (*ρ* = 0)]. The propulsion machinery, for most cells the ATP (adenosine triphosphate)-consuming polymerization of actin filaments and the motor-induced contraction of the actin network, is modeled by a phenomenological equation for the vector field **p**(*x*, *y*; *t*) describing the mean local actin orientation.

The coupling between the *ρ* and **p** fields is motivated by the following biological processes: actin is nucleated close to the membrane (by a cascade of initiators like WASP and Arp2/3) with a rate *β*. On the other hand, existing actin that is polymerizing locally pushes against the membrane and advects it along **p**, with a rate *α*. Furthermore, explicit adhesion, characterized by the local density of adhesive sites *A*(*x*, *y*; *t*), is implemented by making the propulsion strength dependent on the number of formed adhesive bonds, so total advection is given by *αA***p**. The adhesion dynamics is governed by a reaction-diffusion equation, where bonds form with a certain rate depending on *A* and |**p**|, and detach when the substrate deformation exceeds a threshold. The substrate is modeled as a 2D (height-averaged) viscoelastic medium for the displacement field **u**(*x*, *y*; *t*) and the coupling to the cell dynamics is via the traction force the cell exerts on the substrate.

For multiple cells, each cell is described by its own phase field *ρ_i_*. Different cells interact via steric repulsion, preventing overlap, and cell-cell adhesion. The rate of repulsion and cell-cell adhesion is characterized by the parameters *λ* and *κ*, respectively, see Methods.

The model is calibrated for fish keratocytes, crescent-shaped cells of size ~ 20 *μ*m that migrate persistently with a speed of about 0.1 – 0.5 *μ*m/s^51^,^52^. The results are shown in rescaled units: the unit of time is one second and the unit of length one micron. The size of the cells was specified by using circular initial conditions with radii *r*_0_ = 10 or 15. Typical sizes of the studied domain were 100 and 200, and we simulated up to ~60 cells.

We stress that the motion of every cell is self-organized within the proposed modeling framework: symmetric cells are stationary. However, they can be set into motion by perturbations, either by the initial conditions or by cell-cell interactions.

## Results

### Binary interactions of cells

**Figure 1:**
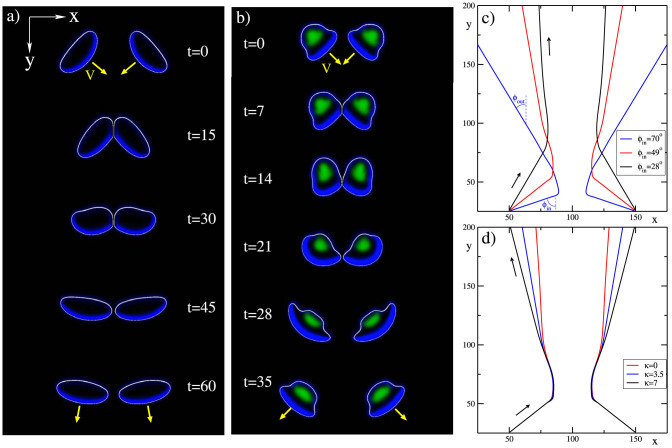
Binary interactions (collisions) of motile cells. (a) Strongly inelastic collision of two canoe-shaped cells (*γ* = 0.5, *σ* = 1.3), leading to an effective alignment of the directions of motion. (b) An almost elastic collision of two bell-shaped cells (*γ* = 0.7, *σ* = 0.6). In (a) and (b), contours of the cells are given in white, the absolute value of the actin orientation in blue and regions with high adhesion in green. The velocities are indicated as yellow arrows. The lateral size is 200 dimensionless units. (c) Effect of the incidence angle on the cells' center of mass trajectories. The red curve corresponds to the snapshots shown in (a). The direction of motion of cells is indicated by the arrows. (d) Effect of adhesion strength *κ* on the cells' center of mass trajectories: increasing adhesion reduces the effective alignment of cells. Initial radius of cells: *r*_0_ = 15, domain size: *L* = 200, periodic boundary conditions. See Supplementary Movies 1 & 2.

Our model reveals a rich phenomenology and dynamics of binary cell interaction. We highlight two distinct cases. Figure 1a) shows the interaction (“collision”) of two cells with “keratocyte-like parameters” (motor asymmetry parameter *γ* = 0.5 and contractility parameters *σ* = 1.3, see Methods). Similar to keratocytes, the cells have a canoe-like shape with a high aspect ratio. They display low intermittent adhesion and move with a constant high speed. The interaction between these cells leads to an effective mutual alignment, that can be considered as a fully inelastic collision^53^. Center of mass trajectories for different incidence angles show that the alignment is more efficient at small incidence angles, Fig. 1c): the smaller the incidence angle, the stronger the cells align upon interaction. In the shown example, the relative change in angles 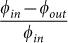 is 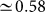 for 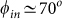 vs. 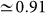 for 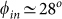. This nonlinear angle dependence is due to the active cell response in the course of collision (coupled reorganization of shape, polarization, adhesion, and substrate deformation). Multiple inelastic collisions between these self-propelled entities lead to mutual alignment of individual cell velocity vectors. In turn, the velocity alignment increases correlations between cell movements and promotes the onset of collective motion, similar to that found in granular-like systems of self-propelled inelastic discs^54^.

Figure 1b) shows the collision of two cells with “fibroblast-like parameters” (*γ* = 0.7 and *σ* = 0.6). For these parameters, well-separated cells are bell-shaped, have strong adhesion (green color) at the rear and a thick region of high actin alignment (blue color) at the front. The cells' velocity can have a small oscillatory component (due to stick-slip dynamics of adhesion, cf. Ref. 45). In the course of interaction, these cells become severely deformed and exhibit intermittent elongated shapes. Strikingly, the cells' collision is almost perfectly elastic: the absolute values of the post-collision angles are very close to the respective incidence angles. Hence, a cluster of such cells should disperse more efficiently than the cells with strongly inelastic interactions, which may be advantageous for searching strategies and covering a surface. However, assemblages of such cells are less prone to collective motion: upon repeated collisions, the directions of motion of the cells stay randomized.

Our model describes some details of the motility machinery (see Methods and Supplementary information). Therefore, it not only captures a variety of different cell types – differing in shape, adhesion/polarization pattern and mode of motion – but in addition allows to relate the observed dynamics to intracellular processes. For example, Figures 1a),b) indicate that decreasing the contractility, i.e. myosin activity or concentration, decreases the inelasticity of collisions and, consequently, the propensity of cells to move collectively.

It is believed that cell-cell adhesion promotes collective movement of cells since the sense of the direction of motion is transferred from one cell to another. Figure 1d) shows, however, the opposite trend: increasing the cell-cell adhesion parameter *κ reduces* the effective alignment. This behavior is reminiscent to that of particle models of collective motion with cohesion^25^. There, the decrease in global orientation with the increase of cohesion is due to the formation of small, short-lived clusters that continuously merge and break up^55^. Decreased cell-cell adhesion was reported to suppress collective motion in cells^5^. However, the cancerous cell lines used could have, in addition to a reduced adhesion, many other cellular parameters affected as well (e.g. elasticity, propulsion strength).

### Transitions between spreading and collective motion

Cells are dynamically bistable: for the same conditions, a cell can either be in a symmetric stationary state – corresponding to a rounded cell spreading on the substrate – or in a polarized moving state^43^,^51^. Experiments on cellular fragments^51^ have shown that, depending on the conditions like incidence angle and speed, motile cells either can set stationary cells into motion or become stopped by them.

**Figure 2:**
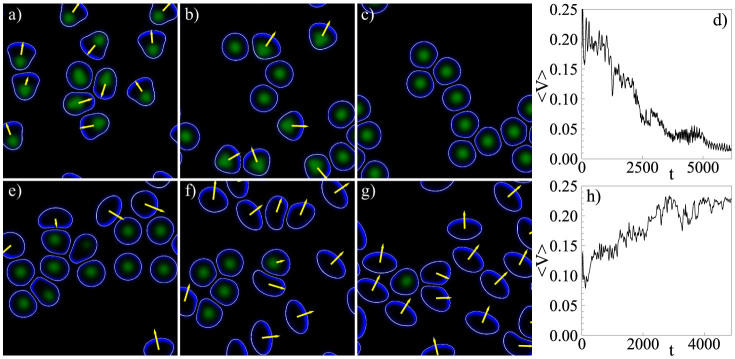
Transitions triggered by cell density and environmental conditions. (a)–(c) Sequence of snapshots illustrating how initially moving cells come to rest and spread on the substrate. (e)–(g) A few motile cells excite the motion of all cells. The values of the parameters are the same as in (a)–(c) except for an increased number of cells and increased value of the parameter *a_nl_* from 1 to 1.1 (cf. the modeling of the adhesive bond formation in the Supplementary Information). Panels (d) and (h) show the average velocity 〈*V*(*t*)〉 for the two scenarios above, respectively. See also Supplementary Movies 3 & 4.

We have investigated the effects of collisions in small cell populations; select results are shown in Fig. 2. Images a)–c) illustrate a scenario where initially moving cells come to rest due to collisions with other cells, see Supplementary Movie 3. Finally, motion ceases and clusters of stationary cells spread on the substrate. Images e)–g) illustrate the opposite trend – for the same parameters as in a)–c), except for a slightly increased cell density and adhesion formation rate *a_nl_*. Here, a few motile cells set all other cells into motion, see Supplementary Movie 4. These two opposite trends can be quantified by calculating the averaged velocity 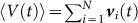, as shown in Fig. 2d) and h), respectively. Note that, as the model has time and length units of one second and a micron, respectively, the average velocities as shown Fig. 2h) correspond to 0.1–0.2 microns per second, well in the range for keratocyte speeds^52^.

The observed behaviors highlight two key phenomena. Firstly, the bistability of the cell polarization dynamics leads to a coexistence of stationary and motile states, as found in experiments. Secondly, that transitions between these states can be triggered by the environmental conditions, interactions, as well as by effective parameters like cell density and collision probability.

### Collective migration

For low cell densities, the collective migration is dominated by binary interactions. Fully inelastic collisions, see Fig. 1a), can lead to the alignment of the migration directions and the onset of collective unidirectional motion. We studied two generic situations. First, a system with periodic boundary conditions, corresponding to a cell population that is far from all boundaries. A similar situation has been realized experimentally in Ref. 7. Second, a circular confined domain where the cells can adhere, surrounded by a region where adhesion to the substrate is suppressed. This geometry was studied experimentally in Refs. 5, 6, where circular domains were prepared by micro-contact printing of adhesive ligands.

**Figure 3:**
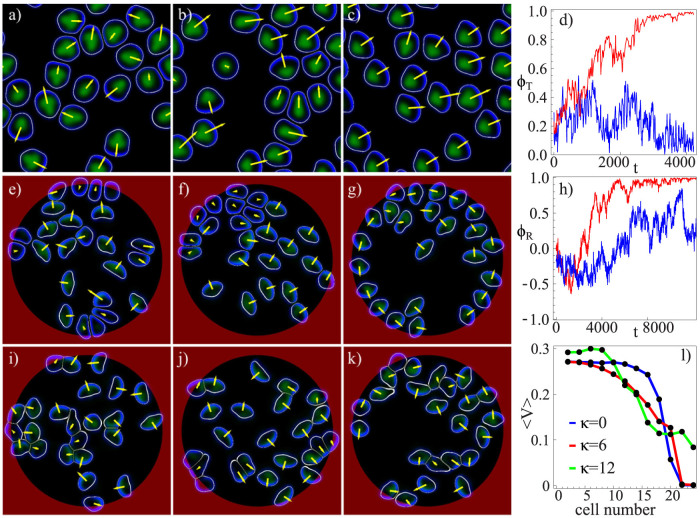
Collective migration of cells. (a)–(c) Emergence of a translational collective migration of 20 cells in a periodic domain without cell-cell adhesion. (d) The order parameter *ϕ_T_* (*t*) for cells without (red, *κ* = 0) and with (blue, *κ* = 6) cell-cell adhesion. Here, the alignment due to inelastic collisions, cf. Fig. 1, leads to a collective unidirectional motion, see Supplementary Movie 5. (e)–(g) Emergence of a rotational collective motion in a circular confined domain. In the red region, the adhesive bond formation to the substrate (parameter *a*_0_, see Supplementary information) is reduced by a factor of 9. See Supplementary Movie 6. (h) The order parameter *ϕ_R_*(*t*) for cells without (red, *κ* = 0) and with (blue, *κ* = 6) cell-cell adhesion. (i)–(k) Adhesion (*κ* = 6) suppresses collective rotational motion, although large fluctuations of the order parameter *ϕ_R_*(*t*), as shown in h), indicate transient collective behavior, see Supplementary Movie 7. (l) Average velocity normalized by the total number of cells *N* for cells moving in a periodic domain for different value of the cell-cell adhesion (*κ*). Initial radius of cells: *r*_0_ = 10, domain size: *L* = 100.

Figure 3a)–c) shows the emergence of translational collective migration in the periodic domain. We defined an order parameter for the translational collective motion via
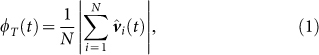
where 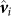 is the unit velocity vector of the *i*-th cell. For large cell numbers, the order parameter will vanish if the velocities are random. It will tend to 1 if all cells are aligned. The red curve in Figure 3d) illustrates the emergence of collective unidirectional motion from an ensemble of cells with initially random directions: after a transient of about *t* = 3000, the order parameter *ϕ_T_* approaches a value of one.

Figure 3e)–g) shows collective motion in the confined circular domain. After a transient, all 24 cells perform a counter-clockwise rotation. Again this can be quantified by introducing an order parameter for rotational collective motion via
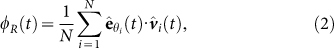
where 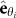 is the unit vector in angular direction of the i-th cell. The red curve in Fig. 3h) shows its evolution for the scenario displayed in e)–f), the final value close to 1 corresponding to counter-clockwise rotation.

In both cases, the collective motion was established after some transient, typically when the cells have migrated a distance of the order of 50–100 times their own size (roughly 3000–4000 dimensionless time units). As a counter example, we simulated cells with elastic collisions [as in Figure 1b)], which did not exhibit any collective migration on the considered time scales (up to 8000 time units). Hence the simple picture of inelastic collisions inducing the transition, deduced from the binary interactions, prevails up to moderate cell densities (volume fraction is about 0.4–0.5). In the circular domain, the interactions of cells with the boundary (depending on parameters and incidence angle, they are reflected or trapped by the boundary, cf.^45^,^47^) forces a transition from translational to rotational collective motion, similar to that observed in Ref. 5.

**Figure 4:**
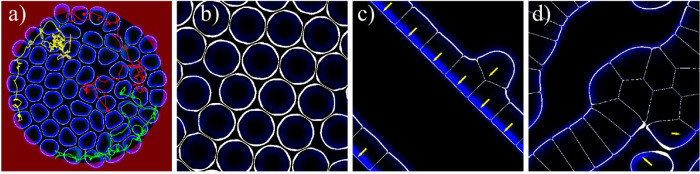
Motion in the regime of high cell density and effect of cell-cell adhesion. (a) Confined high-density state with 61 cells and no cell-cell adhesion (*κ* = 0). Cells compete for voids, thereby moving slowly through the “crowded environment” in a random walk fashion, see Supplementary Movie 8. Color lines show the center of mass trajectories for select cells. (b) Stationary hexagonal arrangement of 22 cells for *κ* = 6. (c) A traveling band of 8 cells for high adhesion, *κ* = 12. It propagates faster than a single cell (cf. the green curve in Fig. 3l), see also Supplementary Movie 9. (d) Clustering of 18 cells due to strong cell-cell adhesion forces (*κ* = 12). A few cells are motile, leave and join the cluster and the cluster changes its shape in time, see Supplementary Movie 10.

Let us now study the effect of cell-cell adhesion, which so far was absent (*κ* = 0). Increasing the cell-cell adhesion parameter to moderate values (*κ* = 6) leads to a break-down of the collectively rotating state, see Fig. 3i)–k), as anticipated from the reduction of the collision inelasticity (cf. section Binary interactions of cells.) This is confirmed by the order parameters – for both geometries studied – as shown by the blue curves in Fig. 3d) and h). Nevertheless, large fluctuations in the rotational order parameter *ϕ_R_* indicate the formation of moving multi-cell clusters, see also Fig. 4c). This state exhibits random reversals of the rotation direction and no trend towards overall collective rotation.

For larger cell densities, cell contacts become protracted and the behavior becomes increasingly dominated by multiple simultaneous cell collisions. Nevertheless, collective motion may still be possible, depending also on cell-cell adhesion. We have found that the average velocity decreases with increasing cell density until a kind of “jamming” transition occurs. This can be inferred from Fig. 3l), where the average velocity as a function of the number of cells in the confined domain is shown. The critical density at jamming slightly depends on the parameters, especially on the cell-cell adhesion: the critical density slightly increases with adhesion strength. Close to jamming, cells compete for voids in the “crowded environment”: individual cells exhibit a wiggling motion in “cages” formed by the other cells, followed by escapes and random walk-like motion as shown by the trajectories of select cells in Fig. 4a). The movement of escaping cells triggers rearrangements of the surrounding cells, as can be inferred from Supplementary Movie 8.

Further consequences of cell interactions are shown in Fig. 4b)–d). For very high densities, all cells stop and form a stationary hexagonal array, see Fig. 4b). At smaller densities but for higher cell-cell adhesion parameter (*κ* = 12), cells gather in traveling bands (phalanges) as shown in Fig. 4c). In a periodic domain this effect leads to collective motion, while the bands break up and reverse in the case of a circular domain. Finally, for high cell-cell adhesion and increased cell density stationary clusters, with a tissue-like arrangement of the cells, form that are surrounded by motile cells which leave/join the clusters in a random fashion, see Fig. 4d).

## Discussion

We have developed a computational model for the collective migration of many self-organized, polarizable and deformable cells. On a single cell level, the model is based on the well-established mechanisms of cell motility accounting for actin polymerization/depolymerization, motor-induced contractility, and adhesive bond formation to a deformable substrate. The admissible ranges of the model parameters are established from available experimental data^43^.

Our study of collective cell motion has reproduced many experimentally observed regimes, from the activation of non-motile cells by moving cells due to steric/adhesive interactions^51^, the emergence of coherently moving^7^ or rotating clusters^5^, to the formation of tissue-like stationary clusters. The model suggested a number of testable predictions. For example, for low cell-cell adhesion, cells move collectively if their interactions are close to inelastic collisions. Increasing the adhesion to moderate values, our study indicated that the collective motion is inhibited due to the formation of short-living clusters of a few cells. Finally, strong adhesion leads to the formation of densely-packed collectively moving bands. These findings provide additional insight into comparative studies of adhesive healthy cells and weakly adhering cancerous cells^5^.

Clearly, the non-trivial binary interaction behavior described here is beyond simple particle-based models: the outcome of a “collision”, which may be elastic or inelastic, is a result of the complex interplay between the cell shapes, their actin polarizations, and adhesions to the deformed substrate. Also the bistability found here is typically not present in other models. For example in the cellular Potts model (as employed in Ref. 5 to describe collective rotational motion) a constant motile force and a certain persistence time of motion are typically used. Thus one expects that our model will be more realistic for non-confluent cells. In contrast, for confluent cell layers, our approach might be too detailed as the individual effects average out. There, the cellular Potts models or coarse-grained continuum approaches may be more suited for the description of basic features. Therefore, our work represents an important bridge from single cell behavior to confluent layer dynamics.

Experimental studies of the “collision” dynamics of two or several cells may yield valuable insight into the onset of collective migration. Binary cell interactions may be further quantified in terms of inelastic collisions^53^. In order to accumulate sufficient statistics on cell collisions, a “cellular collider” is proposed: instead of sampling random (and rare) cell collisions on an unconstrained substrate, the cells may be forced to collide at high rates and at desired angles by micro-contact printing of guiding adhesive ligands patterns. A transition from fully inelastic to almost elastic collisions may indicate an important change in the cell's phenotype: cells colliding inelastically have the propensity to move in groups or to form tissues, whereas elastically colliding cells disperse. Finally, our study in the high density regime may stimulate a better understanding of cancer cell motility and metastasis processes in crowded, high cell density environments.

## Methods

### Description of the computational model

The deformable cell boundary of each cell (*i* = 1 … *N*, where *N* is the total number of cells) is described by a scalar phase field *ρ_i_*:
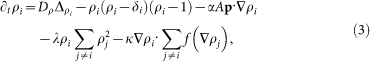
The phase field interpolates smoothly between the cells' interiors (where *ρ* = 1) and the exterior (where *ρ* = 0), the thin transition region being identified with the cell membrane.

Since Eqs. (3) are not conserving the areas of individual cells, the following constraints are introduced to implement the cell volume conservation
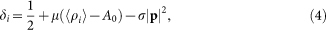
where 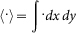, 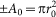 is the initial (2D) cell volume, and *μ* is the stiffness of the constraint. The last term in Eq. (4) models, in a simplified fashion, the effects of actin network contraction^43^. This term also largely controls the shape of individual cells: increasing *σ* makes the cells more compressed in the direction of motion, cf. Figure 1a) and b).

Novel model elements are the two last terms in Eqs. (3): the first one models steric interactions between the cells by penalizing an overlap of the phase fields *ρ_i_* and *ρ_j_* with rate *λ*. The last term models cell-cell adhesion: the field *ρ_i_* is advected along the normal vector to the interface of cell *j* with rate *κ*, which is equivalent to attraction between the cells. The function 
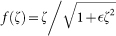
 is used for regularization.

Finally, the cell's propulsion is modeled in Eqs. (3) by the advection (with rate *α*) along the coarse-grained averaged actin polarization, which is described by the vector field **p**. Its direction corresponds to the mean actin orientation and its modulus to the mean degree of orientation and actin density. The actin polarization dynamics is coupled to the phase fields of all cells and given by
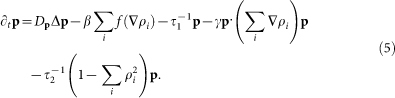
In order of appearance, the terms on the r.h.s. describe diffusion/elasticity of actin, creation of actin at the cell membrane (with polymerization rate *β*; *f* is again used for regularization), depolymerization (with rate *τ*_1_), front/tail asymmetry (induced by myosin motors, parameter *γ*), and suppression of actin in the outside of the cells.

The dynamics of the adhesive bonds, *A*, promoting cell advection [cf. 3rd term on r.h.s of Eq. (3)], is modeled by a reaction-diffusion equation:
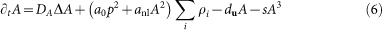
with on the r.h.s. diffusion *D_A_*, linear (*a*_0_) and nonlinear (*a_nl_*) attachment rates, a step-like detachment function when substrate displacement exceeds a threshold (*d***_u_** = 1 for |**u**| > *U*_0_, 0 otherwise) and an excluded volume term. In turn, the substrate displacements are caused by the traction forces induced by all cells. The substrate is modeled as a viscoelastic solid (Kelvin-Voigt model) in thin layer approximation, with the displacements **u** governed by^47^
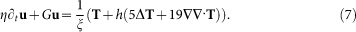
Here *G* is the modulus of the substrate and *η* accounts for dissipation in bond rupture. **T** is the sum of the traction forces *T_i_* exerted by all cells
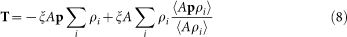
where the first term is the counterforce of actin pushing, and the second is due to friction with the substrate, see Ref. 47. *ξ* characterizes the efficiency of the force transmission. Since the cells are self-propelled, the total traction is zero, 〈**T**〉 = 0.

The main parameters are summarized in table 1, all values are given in the Supplementary Information. There, also a sensitivity study of the model results on select parameters can be found.

## Author Contributions

J.L., F.Z. and I.S.A. designed and performed the study. J.L., F.Z. and I.S.A. wrote the paper.

## Supplementary Material

Supplementary Information Supplementary Movie 1. Inelastic collision of cells

Supplementary Information Supplementary Movie 2. Elastic collision of cells

Supplementary Information Supplementary Movie 3.Transition from moving to stationary cells

Supplementary Information Supplementary Movie 4. Transition from stationary to moving cells

Supplementary Information Supplementary Movie 5. Translational collective migration

Supplementary Information Supplementary Movie 6. Rotational collective migration

Supplementary Information Supplementary Movie 7. Suppression of rotational collective migration by cell-cell adhesion

Supplementary Information Supplementary Movie 8. Cells competing for voids

Supplementary Information Supplementary Movie 9. Traveling band of cells

Supplementary Information Supplementary Movie 10. Clustering of cells

Supplementary Information Supplementary notes and table

## Figures and Tables

**Table 1:** The main parameters in the multi-cell phase field model. For an extensive list and description, see the Supplementary Information

Actomyosin parameters		
parameter	value	description
*α*	2–5	propulsion by actin polymerization
*β*	*α*/2	actin polymerization rate
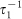	0.1	degradation rate of actin
*γ*	0–1	motor-induced front/back asymmetry
*σ*	0–1.5	actomyosin contraction
Cell-cell interaction parameters		
*λ*	30	steric repulsion strength
*κ*	0–12	cell-cell adhesion strength
