# Supplementary material for: Collisions of deformable cells lead to collective migration
Source: Sci Rep. 2015 Mar 17;5:9172. doi: 10.1038/srep09172 (PMC4361886; doi:10.1038/srep09172)
Supplement: Supplementary Information — Supplementary notes and table [file srep09172-s11.pdf]

Supplementary information:
Collisions of deformable cells lead to collective migration

Jakob Löber[1], Falko Ziebert[2,3] & Igor S Aranson[4,5]

[1] *Institut für Theoretische Physik, Hardenbergstrasse 36, EW 7-1, Technische Universität Berlin, 10623 Berlin, Germany*

[2] *Physikalisches Institut, Albert-Ludwigs-Universität Freiburg, Hermann-Herder-Strasse 3, 79104 Freiburg, Germany*

[3] *Institut Charles Sadron, CNRS-UPR22, 23 rue du Loess, 67034 Strasbourg Cedex 2, France*

[4] *Materials Science Division, Argonne National Laboratory, 9700 S. Cass Avenue, Argonne, IL 60439, USA*

[5] *Engineering Sciences and Applied Mathematics, Northwestern University, 2145 Sheridan Road, Evanston, IL 60202, USA*

Supplementary Notes 1. Model

We generalized the model for a single cell developed by us in Refs.[1–3] to an arbitrary number of cells. As new effects, the model includes steric repulsion (with repulsion strength λ) and cell-cell adhesion (with adhesion strength κ).

The single cell model is based on four continuous, two-dimensional fields:

1. The phase field $\rho(x, y; t)$ used to track the moving cell interface. It attains the value $\rho = 1$ inside the cell and $\rho = 0$ outside the cell, with a smooth transition region between the two, defining the position of the cell interface.

2. The mean actin orientation ('polarization') inside the cell, given by the vector field $\mathbf{p}(x, y; t)$ with two vector components. Its direction denotes the mean orientation and its absolute value the degree of ordering.

3. The density of formed adhesive bonds between the cell and the substrate denoted by $A(x, y; t)$.

4. The displacement field $\mathbf{u}(x, y; t)$ of the substrate (in thin layer approximation) on which the cell is sitting with two vector components.

In case of multiple cells, every cell is described by its own phase field ρ_i, $i = 1, \ldots, N$, with N the total number of cells. The other fields $\mathbf{p}$, A and $\mathbf{u}$ are shared by all cells since $\mathbf{p}$ and A are zero outside each cell and all cells move on one and the same substrate.

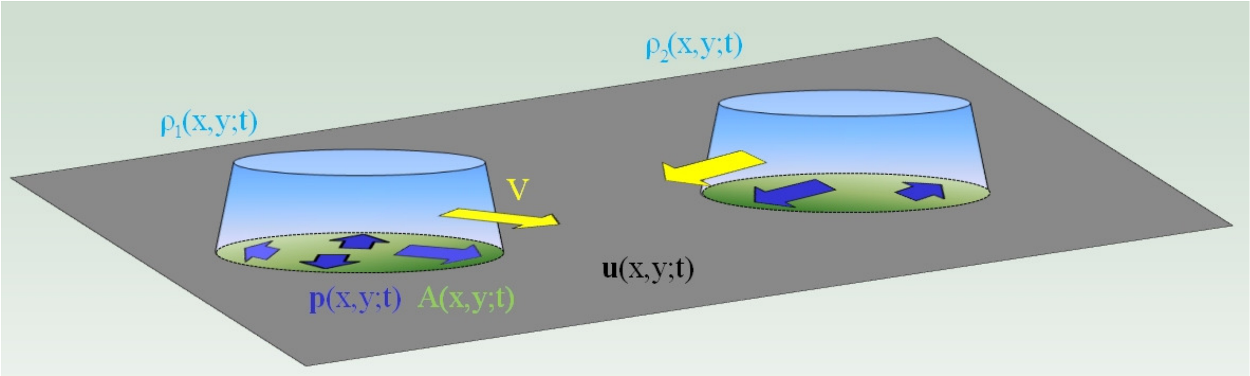

Figure S1: **Schematics of two cells crawling on the substrate**. The shape of cells are described by the order parameter ρ. Cells migrate with the velocity $\mathbf{V}$. $\mathbf{p}$ and A are averaged actin polarization and concentration of adhesive ligands respectively, and $\mathbf{u}$ is the substrate displacement.

The phase field ρ_i of the ith cell is governed by

$$\begin{aligned} \partial_t \rho_i &= D_\rho \Delta \rho_i - \rho_i (\rho_i - \delta_i)(\rho_i - 1) - \alpha A \mathbf{p} \cdot \nabla \rho_i \\ & -\lambda \rho_i \sum_{j \neq i} \rho_j^2 - \kappa \nabla \rho_i \cdot \sum_{j \neq i} f(\nabla \rho_j), \end{aligned} \tag{S1}$$

with

$$\delta_i = \tfrac{1}{2} + \mu (\langle \rho_i \rangle - A_0) - \sigma |\mathbf{p}|^2, \tag{S2}$$

where $\langle \cdot \rangle = \int \cdot \, dx \, dy$ denotes the integral over the whole computational domain. The first line of Eq. (S1) is the single cell model as discussed previously[1–3] and the second line contains cell-cell interactions. The two first terms on the r.h.s. describe the phase field interface: the first term with Laplacian Δ defines the interface width proportional to $\sqrt{D_\rho}$, the second term denotes the restoring force of the phase field potential. The feedback implemented via the parameter δ accounts for volume conservation (second term in Eq. (S2), with stiffness μ) and acto-myosin contractility

(via parameter σ). The third term on the r.h.s. describes the main propulsion mechanism of the cell: the interface is advected along the mean actin orientation – provided that polymerizing actin $\mathbf{p}$ is present and linked to the substrate via adhesive bonds A.

The second line of Eq. (S1) describes the two predominant cell-cell interactions between cell i and all other cells $j \neq i$: steric repulsion and cell-cell adhesion. Steric repulsion between two cells i and j is modelled as a quadratic pair potential $V_{ij} = \frac{\lambda}{2}\rho_i^2\rho_j^2$ in the phase fields which leads to a term $\partial_t \rho_i \propto -\frac{\delta}{\delta\rho_i}\sum_{i\neq j} V_{ij}$ for the dynamics of ρ_i. The potential energy V_{ij} is large if the cells i and j overlap, and zero if they do not overlap. Because the phase field dynamics Eq. (S1) minimizes the total energy, this term penalizes overlapping cells and results in cells which do not overlap. Cell-cell adhesion is modeled by advection of cell i towards cell j along the normal to the boundary of cell j, with rate κ. To prevent numerical instabilities related to very steep gradients of ρ_j, we normalized the gradient in ρ_j via a function $f(\zeta) = \frac{\zeta}{\sqrt{1+\epsilon\zeta^2}}$, implementing saturation. Summing up cell-cell adhesion from all cells, this amounts to the last term in Eq. (S1).

The actin polarization is modeled as previously[3], except that now the phase fields of all cells contribute:

$$\partial_t \mathbf{p} = D_{\mathbf{p}}\Delta\mathbf{p} - \beta\sum_i f(\nabla\rho_i) - \tau_1^{-1}\mathbf{p} - \gamma\mathbf{p}\cdot\left(\sum_i \nabla\rho_i\right)\mathbf{p} - \tau_2^{-1}\left(1 - \sum_i \rho_i^2\right)\mathbf{p}\,. \quad \text{(S3)}$$

In order of appearance, the terms on the r.h.s. describe diffusion/elasticity of actin, creation of actin at the cell membrane with polymerization rate β, depolymerization with rate τ_1, a term responsible for a front/tail asymmetry induced by myosin motors with parameter γ as derived in [1], and suppression of actin outside of all cells. In the second term on the r.h.s, we again used the function $f(\zeta) = \frac{\zeta}{\sqrt{1+\epsilon\zeta^2}}$ to prevent excessive creation of $\mathbf{p}$ due to steep phase field gradients.

The cell-substrate adhesion bonds are modeled as previously[2]

$$\partial_t A \;=\; D_A \Delta A + \left(a_0 p^2 + a_{\rm nl} A^2\right) \sum_i \rho_i - d_{\mathbf{u}} A - s A^3 \,. \qquad \text{(S4)}$$

In order of appearance, the terms on the r.h.s describe: i) diffusion, ii) linear attachment of the cell to the substrate with rate a_0, iii) nonlinear attachment with rate a_{nl} due to collective adhesion effects motivated by the fact that it is easier to form a bond if another one exists close-by, iv) detachment, with a rate modeled via a step-like detachment function $d_{\mathbf{u}} = d\left(|\mathbf{u}|\right)$, which is nonzero when the substrate displacement exceeds a threshold, and v) an excluded volume term, implementing saturation of A.

Finally, the substrate is modeled as a viscoelastic solid (Kelvin-Voigt model) in thin layer approximation, with the displacements $\mathbf{u}$ governed by[3]

$$\eta \partial_t \mathbf{u} + G\mathbf{u} \;= \tfrac{1}{\xi}\left(\mathbf{T} + h\left(5\triangle \mathbf{T} + 19\nabla\nabla\cdot\mathbf{T}\right)\right). \qquad \text{(S5)}$$

Here G is the elastic (shear) modulus of the substrate and η its viscosity.The vector field $\mathbf{T}$ with two vector components is the sum of the traction force $\mathbf{T}_i$ exerted by each cell

$$\mathbf{T} \;= \textstyle\sum_i \mathbf{T}_i = -\xi A\mathbf{p}\sum_i \rho_i + \xi A \sum_i \rho_i \frac{\langle A\mathbf{p}\rho_i\rangle}{\langle A\rho_i\rangle}. \qquad \text{(S6)}$$

The first contribution is the negative of the actin propulsion force and the second one is associated to friction[3]. Note that the integral of the traction force $\mathbf{T}_i$ over the whole computational domain of each cell is zero, $\langle \mathbf{T}_i\rangle = 0$, as it should be.

In conclusion, the cells interact via repulsion with parameter λ, cell-cell adhesion with parameter κ, and the complex but short-ranged interaction mediated by the substrate.

Supplementary Notes 2. Numerical solution and analysis

To solve the equations numerically, we use a highly parallel algorithm implemented on GPUs using CUDA. The code can handle an arbitrary number of cells with single or double precision. Periodic boundary conditions in x- and y-direction are applied for all fields, such that a pseudo spectral approach using the batch Fast Fourier Transform implemented with CUFFT can be used. We typically choose a resolution of 512 up to 2048 Fourier modes for a domain size of $L = 100$ up to $L = 200$. For comparison, the initial cell radius is typically chosen between $r_0 = 10$ and $r_0 = 15$. Sums over phase fields as e.g. $\sum_{j=1}^{N} \rho_j^2$ and $\sum_{j=1}^{N} \nabla \rho_j$ are computed with customized CUDA kernels. The center of mass of each cell has been computed as described in Ref. [4] to track the position of each cell over time, from which its velocity over time is readily determined in finite difference approximation. Mean velocities as well as order parameters over time are obtained by summing over all cells. The time-averaged mean velocity is determined by a subsequent averaging over all time steps for sufficiently long time intervals to diminish the contribution of initial transients.

Supplementary Notes 3. Parameters of the model

A list of the parameters of the model, their standard values used in numerical simulations, and a description of the associated physical effects can be found in Table S1. In addition, the stiffness of the volume constraint was fixed at $\mu = 0.1$, actin suppression outside of cells at $\tau_2^{-1} = 0.4$, and in $f(\zeta)$, cf. Eq. (S3), $\epsilon = 37.25$ was used.

Although there is a large number of parameters, the model is not sensitive to all of them. This is

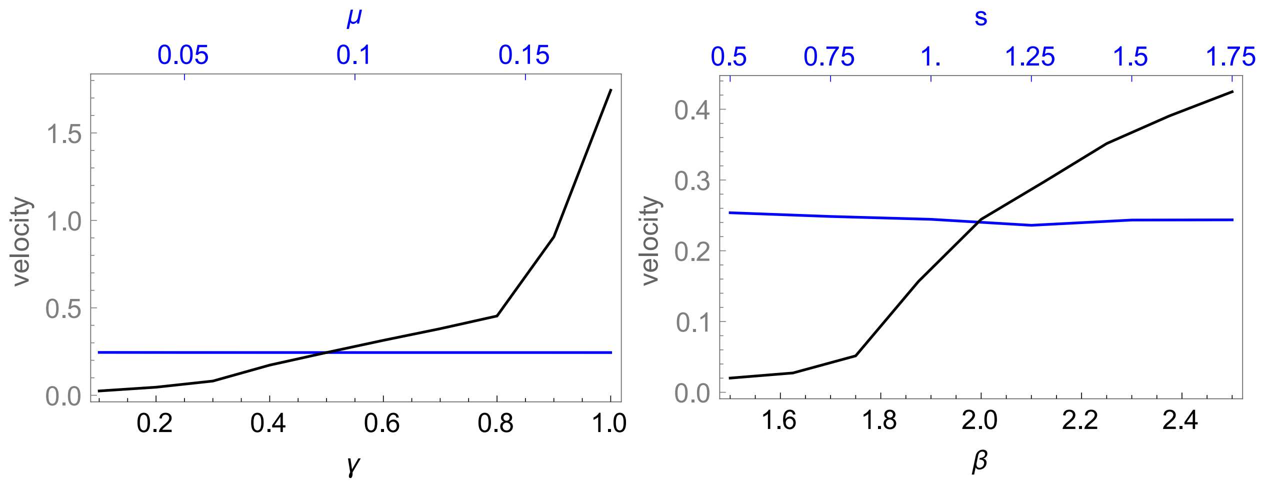

Figure S2: **Sensitivity of a single cell's time-averaged velocity with respect to selected parameters.** Left: Varying γ changes the front/tail asymmetry induced by myosin motors and has a dramatic effect on cell velocity, while it is almost independent of μ characterizing the stiffness of the volume constraint. Right: Increasing the polymerization rate at the cell membrane β accelerates the cell, but changing the nonlinear detachment rate of adhesive bonds has a negligible effect on cell velocity.

exemplified in Fig. S2: there it is shown that the propulsion related parameters (here the motor-induced front-tail asymmetry quantified by γ and the actin creation rate at the membrane, β) determine the cell velocity, while other parameters like the stiffness of the volume constraint, μ, or the saturation value of adhesive bonds, determined by s, have practically no influence. Similar studies were carried out in earlier works on single cells [1,2]. The most important parameters have been discussed and tabled in the main manuscript.

parameter	value	description
New interaction parameters		
λ	30	steric repulsion
κ	0-12	cell-cell adhesion
Phase field and actin dynamics, cf. Ref.[1]		
α	2-5	propulsion rate due to actin polymerization
β	$\alpha/2$	actin nucleation/polymerization rate
τ_1^{-1}	0.1	degradation rate of actin
γ	0 - 1	head/tail asymmetry induced by motors
σ	0 - 1.5	contraction induced by motors
D_ρ	1	determines width of the diffuse interface
$D_{\mathbf{p}}$	0.2	diffusion of actin
Adhesion to the substrate, cf. Refs. [2,3]		
a_0	0.01 - 0.09	linear attachment rate
a_{nl}	1 - 1.2	nonlinear attachment rate
d	1.0	detachment rate of adhesive bonds
U_c	$\sqrt{0.2}$	critical substrate stretch for bond rupture
s	1	excluded volume of adhesive bonds
D_A	1	diffusion of adhesive bonds
G	0 - 0.3	elastic modulus of the substrate
η	10/3	substrate viscous relaxation rate
h	0.1	substrate thickness

Supplementary Table S1: **Parameters of the model.**

Descriptions of Supplementary Movies.

In all movies, phase field contours are shown in white, the absolute value of the actin orientation in blue and regions with high adhesion in green.

Supplementary Movie 1. Inelastic collision of cells

A strongly inelastic collision of two canoe-shaped cells, leading to an effective alignment of the directions of motion, cf. Fig.1a) in main.

Supplementary Movie 2. Elastic collision of cells

An almost elastic collision of two bell-shaped cells, cf. Fig.1b).

Supplementary Movie 3. Transition from moving to stationary cells

Initially moving cells come to rest and collect in stationary clusters, cf. Fig.2a)-c).

Supplementary Movie 4. Transition from stationary to moving cells

Initially, only few cells move, while cells which adhere strongly to the substrate (those with green spots inside) are stationary. Repeated collisions between moving and stationary cells set all cells into motion, cf. Fig.2e)-g).

Supplementary Movie 5. Translational collective migration

Alignment of propagation directions due to collisions between cells in a domain with periodic boundary conditions. Cells do not adhere to each other. Cf. Fig.3a)-c).

Supplementary Movie 6. Rotational collective migration

Emergence of rotational collective motion in a circular confined domain (in the red region, the nonlinear adhesive bond formation to the substrate is reduced by a factor of 9). Cf. Fig.3e)-g).

Supplementary Movie 7. Suppression of rotational collective migration by cell-cell adhesion

Adhesion between cells prevents the emergence of collective rotational motion, cf. Fig.3i)-k).

Supplementary Movie 8. Cells competing for voids

Confined high-density state without cell-cell adhesion. Cells compete for voids, thereby moving slowly through a “crowded environment” in a random walk fashion, cf. Fig.4a).

Supplementary Movie 9. Traveling band of cells

A band of cells strongly adhering to each other is moving in a domain with periodic boundary conditions, cf. Fig.4c).

Supplementary Movie 10. Clustering of cells

Clustering of cells due to strong cell-cell adhesion forces. A few cells are motile and the cluster changes its shape in time. Cells leave and join the cluster. Cf. Fig.4d).

Supplementary References

1. Ziebert, F., Swaminathan, S. & Aranson, I. S. Model for self-polarization and motility of keratocyte fragments. *J. Royal Soc. Interface* **9**, 1084 (2012).

2. Ziebert, F. & Aranson, I. S. Effects of adhesion dynamics and substrate compliance on the shape and motility of crawling cells. *PLoS ONE* **8**, e64511 (2013).

3. Löber, J., Ziebert, F. & Aranson, I. S. Modeling crawling cell movement on soft engineered substrates. *Soft Matter* **10**, 1365–1373 (2014).

4. Bai, L. & Breen, D. Calculating center of mass in an unbounded 2d environment. *Journal of Graphics, GPU, and Game Tools* **13**, 53–60 (2008).
